# Effect of mailed feedback on drug prescribing profiles in general practice: a seven-year longitudinal study in Storstrøm County, Denmark

**DOI:** 10.3109/03009734.2010.487165

**Published:** 2010-10-27

**Authors:** Keld Vægter, Rolf Wahlström, Hans Wedel, Kurt Svärdsudd

**Affiliations:** ^1^Uppsala University, Department of Public Health and Caring Sciences, Family Medicine and Clinical Epidemiology, UppsalaSweden; ^2^Unit of Continuous Medical Education in General Practice (FUAP), Health Department, Storstrøm County, Nykøbing FDenmark; ^3^Centre for Development and Research in Primary Care in Sörmland, EskilstunaSweden; ^4^Division of Global Health (IHCAR), Department of Public Health Sciences, Karolinska Institute, StockholmSweden; ^5^Nordic School of Public Health, GothenburgSweden

**Keywords:** Drug prescribing, general practice, mailed feedback

## Abstract

**Background:**

Whether written feedback on drug prescribing in general practice affects prescribing habits is controversial. Most short-term studies showed no effect. However, the issue has not been tested in long-term studies involving the local general practitioner community.

**Aims of the study:**

To assess whether prescribing levels in general practice are affected by long-term, unsolicited, systematically repeated, mailed feedback.

**Methods:**

Each of the 94 general practices in Storstrøm County, Denmark, received semi-annual, mailed feedback about their prescribing volumes and costs within 13 major drug groups, in relation to the levels for all the other 93 practices over a 7-year period in a project initiated by the local general practitioner association. Data on the number of defined daily doses (DDDs) prescribed per 1000 listed patients in each practice per 6-months, and practice characteristics, were obtained from the Pharmaceutical Database at the County Health Department.

**Results:**

There was a large variation in drug prescribing volume between practices, but little within-practice variation over time. After adjustments for the influence of practice size and other potential outcome-affecting variables, there was no evidence of a general change of prescribing volume over time, no change among practices with a high or a low prescribing level, and no significant change within the various drug groups.

**Conclusions:**

We found no significant effects on prescribing levels of mailed feedback, even when repeated semi-annually during 7 years and initiated by the local general practitioner community.

## Introduction

Drug prescribing in general practice is subject to attention from all interested parties. In Scandinavia, the majority of prescriptions, regardless of medical specialty, are issued by general practitioners (GPs) (1,2). Significant variations in prescribing habits among GPs have been shown (3,4), which to a certain extent may be explained by variations in demographic factors, including patient age, sex, and diagnostic panorama (‘case mix’). There may, however, be as yet unidentified additional reasons for these variations, such as the prescribing habits of individual GPs.

The ‘optimum’ prescribing profile for a practice is difficult to define. A basic step in the drug prescribing quality assessment process is to become aware of one's own prescribing profile. This may be accomplished by displaying the prescribing profile of a given practice as compared to the variations among all practices in the area, for instance by mailed feedback. Whether this undertaking is enough to start a perpetual rational drug therapy review on the practice level has been debated (4,5). The effect of mailed feedback is controversial. In a number of studies no effect was found (6–9), whereas others have reported limited positive effects in general practice of postal feedback of prescribing profiles for selected drug groups combined with treatment recommendations (10,11).

In 1991 an initiative was launched to improve drug prescribing among GPs in the former Storstrøm County, Denmark. During fourteen 6-month periods, mailed feedback to the individual practices was used to encourage GPs to review their drug therapy profile and, if needed, reconsider their prescribing practices. In this report the effect of this unsolicited, semi-annually mailed feedback on intra- and inter-practice variations in drug prescribing was assessed.

## Material and methods

### Setting

The former Storstrøm County is today part of the larger administrative unit Region Sjælland. At the time of the study it included the southern part of Sjælland, the islands of Falster and Lolland, and a few other minor islands, and had 257,000 residents. The area is mainly rural with a few small towns and was served by 166 general practitioners distributed across 94 practices.

In Denmark the general practitioners are private contractors to the County Health Authority, each taking care of approximately 1500 listed patients. Each practice has a specific identification number (PIN) within the National Health Insurance system. All relevant information related to administration and fees in the practices, such as patient demographics, prescriptions (obtained from the Danish Medicines Agency), referrals, and specific services and treatments performed in the practice, is registered in the local County Health Insurance database.

Traditionally, most practices in primary health care in Denmark have been ‘solo’ practices (run by one GP), but in recent decades the formation of group practices has become increasingly common. In group practices it is not possible to identify the individual GP's contribution to the common prescribing profile, since the PIN refers to the practice as a whole. The population of listed patients in the practice system is stable, with an average annual change between practices of less than 10%. The differences between practice patient populations in terms of age and gender are small (personal communication from the Pharmacoeconomic Division, Danish Medicines Agency).

### Data collection

All prescriptions filled at Danish pharmacies, reimbursed as well as non-reimbursed, are registered in a database at the Danish Medicines Agency by practice PIN code and anatomical therapeutic chemical (ATC) code (12). The registration is almost 100% complete. All prescriptions analysed in this report were fully reimbursed.

In 1991 the first steps were taken to establish a ‘GP Quality Unit’ by collaboration between representatives from general practice and officials from the health administration within the Health Department of Storstrøm County. The aim was to encourage a review among GPs of their prescribing habits in order to improve and enhance rational drug therapy. To visualize differences in prescribing habits and to trigger the awareness of the GPs, prescribing data on reimbursed pharmaceuticals with the ATC codes A02 (antacids), A10 (anti-diabetes drugs), C01 (cardiac drugs), C03 (diuretics), C07 (beta-blockers), C08 (calcium channel-blockers), G03 (reproduction hormones), J01 (anti-bacterial drugs for systemic use), M01 (non-steroid anti-inflammatory drugs), N02 (analgesics), N05 (neuroleptic drugs), N06 (psycho-analeptic drugs), and R03 (anti-asthma drugs) were extracted from the County Health Insurance database, presented in charts, and mailed to each practice every 6 months. No intervention other than the mailed feedback was made.

**Figure 1.**
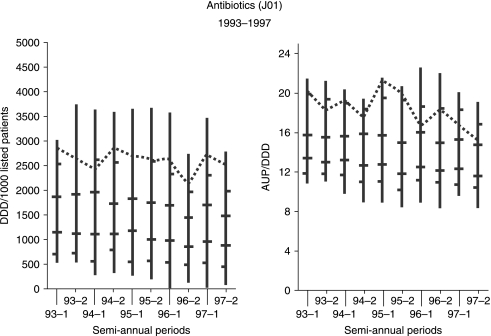
Example of feedback information sheet. The left-hand panel presents data regarding the amount of drugs, in this case antibiotics, prescribed. The right-hand panel presents cost data. The dotted line represents the prescribing level of a specific practice relative to all practices in the county. The vertical scale corresponds to the 5th, 25th, 75th, and 95th percentiles of the distribution across all practices. DDD = defined daily doses; AUP = costs in Danish kroner per DDD.

The feedback diagrams illustrated the prescribing levels of each of the 13 drug groups as number of defined daily doses (DDD) per 1000 listed patients and the practice's percentile position within the distribution across all practices in the county. The corresponding information on costs per DDD prescribed by the practice was presented in a similar way. An example of the feedback diagrams is shown in Figure 1. Every 6 months new data were added to the charts and mailed to the practices.

If this type of feedback works, the anticipated effect would be a clustering of prescribed DDDs towards the mean, i.e. a smaller dispersion between practices and a tendency towards instability of individual practice prescribing patterns over time owing to changing habits. Since the initiative for political reasons was launched full scale simultaneously in all practices throughout the county, no control group was available. Therefore, the prescribing habits of all practices were followed through the study period.

Approval from an ethics committee was not needed since the project did not include direct patient involvement, and no classified information that could reveal patient identity was handled.

### Statistical considerations

Statistical analyses were performed using the SAS software, version 9.1 (13). Data were complete. Simple (crude) differences between groups regarding continuous variables were tested with Student's *t* test and differences in proportions with the chi-square test.

During the observation period 1992–1998 a few new practices were established. This might have given rise to false low values regarding the numbers of prescribed DDD/1000 patients in the opening period. To avoid this problem, data from the first 6-month period of new practices were excluded.

In order to discover and test changing habits four methods were employed. The first focused on intra-practice variation of prescribing habits, where variation would increase in case of changing habits. The prescribing data constitute a time-series of prescriptions issued by the same GP population. The resulting DDDs may therefore be auto-correlated, i.e. the value for a specific 6-month period predicts, to a certain extent, the value of the next period. To overcome this problem the SAS procedure ‘autoreg’ was used to diagnose and to adjust for auto-correlation. The adjustment included the nearest three 6-month periods, as these were significantly auto-correlated. The resulting adjusted measure of variability, mean square error (MSE), may be regarded as a variance adjusted for prescribing trend across time. We used the square root of this measure, which is equivalent to a standard deviation unit, as a measure of prescribing variability within practices over time.

The second method was based on the so-called ‘folding rule’ regression analysis. The study period was divided in two sub-periods, and a regression line of prescribed DDDs per 1000 listed patients over time was calculated for each practice and for each sub-period, much like a folding rule. The meeting-point, or intersection, between the lines was successively moved across the study period. A significant difference in regression coefficient (‘leaning’) between the two lines would then indicate a change of subscribing habit. In this way the existence of a systematic deviation towards the central part of the prescribing distribution might be detected.

Thirdly, the change in the distribution of DDD per 1000 listed patients across the fourteen 6-month periods was tested. If a change of habit had occurred, the distribution would be narrower towards the end of the study period than in the beginning.

Fourthly, scatter plots of the prescription volumes for each practice over time were produced and scrutinized for change of trend.

Univariate and multivariate linear regression analyses were used to analyse the influence of various potential determinants, such as age and sex of the listed patients (‘case mix’), age and sex of the GPs, practice type, and number of years of experience as a GP, on prescribing variation, measured as mean error, or on practice regression coefficients. All tests were two-tailed. The level of significance was set at *P* < 0.05.

## Results

### Characteristics of the study population

Study population characteristics are shown in Table I. Fifty-four practices were solo practices, and 50 were group practices with an average of 3.2 GPs per practice. The 166 GPs were on average 50 years old (interquartile range 46–54 years), 81% were men, mean number of years as a GP was 14.5 (interquartile range 8–20 years). There were no significant differences in the distribution of age, sex, and GP experience between GPs in solo versus group practices.

**Table I.** Characteristics of the study population.

	Solo practices			Group practices			Total		
	*n*	Mean or %	95% CI	*n*	Mean or %	95% CI	*n*	Mean or %	95% CI
*n*	54	57.4		40	42.6		94	100.0	
Age, years	54	50.3	48.7–51.9	112	49.9	48.7–51.1	166	50.0	49.1–51.0
Male physicians, %	46	85.2	75.4–95.0	88	78.6	70.9–86.3	134	80.7	74.7–86.8
No. of GPs in practice	54	1.0	–	112	3.2	2.9–3.4	166	1.77	1.54–1.99
No. of years as a GP	54	15.1	13.0–17.1	112	14.3	12.8–15.7	166	14.5	13.4–15.7

CI = confidence interval; GP = general practitioner.

### Prescribing habits over time

**Figure 2.**
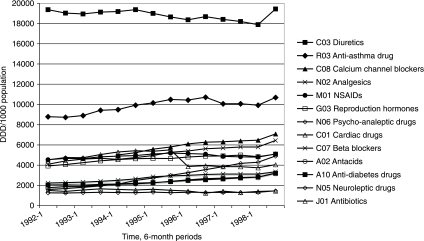
Levels of prescribed defined daily doses (DDDs) per 1000 population per 6 months by drug group. NSAID = non-steroid anti-inflammatory drug.

The overall prescribing rate of the 13 drug groups studied increased from 64,870 DDDs/1000 patients in the first 6 months of 1992 to 70,360 DDD/1000 listed subjects in the last 6 months of 1998, an annual increase rate of 915 DDD/1000 listed subjects. The trends in prescribing rate for the various drug groups are shown in Figure 2. The number of DDDs per 1000 listed subjects prescribed during the study period increased significantly for antacids (A02), anti-diabetes drugs (A10), beta-blockers (C07), calcium channel-blockers (C08), reproduction hormones (G03), analgesics (N02), psycho-analeptic drugs (N06), and anti-asthma drugs (R03), while it decreased for cardiac drugs (C01) and diuretics (C03), and remained unchanged for antibiotics (J01), non-steroid anti-inflammatory drugs (M01), and neuroleptic drugs (N05).

### Prescribing variation among practices

The variations between practices in amount of prescribed drugs within the various ATC groups are presented in Table II as semi-annual mean DDD/1000 listed patients, the 95th and the 5th percentile of the DDD distribution across practices, and the ratio of these two. There were fairly large variations in prescribing level between the practices, as reflected by the 95th to 5th percentile ratios. The largest differences were seen for neuroleptic drugs (ratio 6.0) and the smallest for anti-asthma drugs (ratio 2.5).

**Table II.** Variation of drug prescribing habits, measured as mean standard deviation of prescribed DDD/1000 patients across the follow-up period.

			Percentiles		Ratio 95th/5th	Root MSE		
Drug group	ATC code	Semi-annual mean^a^	95th	5th		Mean	95% CI	Range
Antacids	A02	2475.59	4377.0	1137.4	3.8	0.24	0.21–0.26	0.03–0.66
Anti-diabetes drugs	A10	2460.11	3889.0	1304.4	3.0	0.30	0.27–0.33	0.03–0.73
Cardiac disease drugs	C01	4689.73	8289.7	1981.4	4.2	0.30	0.26–0.34	0.03–1.08
Diuretics	C03	19315.90	31723.8	10143.7	3.1	0.23	0.20–0.25	0.01–0.77
Beta-blockers	C07	2845.88	4695.4	1366.0	3.4	0.25	0.22–0.28	0.02–0.81
Calcium channel-blockers	C08	6123.94	10276.1	2747.2	3.7	0.20	0.18–0.22	0.01–0.61
Sex hormones	G03	4642.50	7545.8	2089.0	3.6	0.22	0.20–0.24	0.03–0.65
Antibiotics	J01	1433.18	2481.2	645.8	3.8	0.25	0.23–0.28	0.01–0.60
NSAIDs	M01	5107.58	8171.3	2854.1	2.9	0.25	0.22–0.27	0.01–0.63
Analgesics	N02	5374.84	9979.6	2416.6	4.1	0.20	0.17–0.22	0–0.55
Neuroleptics	N05	1321.11	2852.4	474.5	6.0	0.23	0.20–0.26	0.01–0.90
Anti-depressants	N06	3139.88	4501.1	1494.0	3.6	0.21	0.19–0.23	0.02–0.51
Anti-asthma drugs	R03	9851.54	14575.7	5900.0	2.5	0.28	0.25–0.31	0–0.76

^a^DDD/1000 patients.

ATC = anatomical therapeutic chemical; CI = confidence interval; DDD = defined daily doses; NSAIDs = non-steroid anti-inflammatory drugs; MSE = mean square error.

There was little variation over time within prescribing practices, as reflected by the root of the MSE, a measure of the mean prescribing standard deviation adjusted for the increasing or decreasing trend line over the fourteen 6-month periods (Table II). The root MSE was 0.20 units, one-fifth of a standard deviation, with a 95% confidence interval (CI) of 0.18–0.23 for all drug groups combined after adjustment for auto-correlation, indicating only minute deviations from the trend line. Among the individual drug groups, analgesics (N02) and calcium channel-blockers (C08) had the lowest variability and anti-diabetes drugs (A10) the highest. The within-practice deviation range was 0.01–1.08 for individual ATC groups.

In the folding rule regression analysis across all practices there was no evidence of a deviation of high or low prescribers towards the mean for all GP practices, nor was there any significant change in the DDD per 1000 listed subjects distribution across time when adjusting for the increasing mean. Scrutiny of practice-specific trend lines for the prescribing levels of the various ATC groups gave no evidence that high or low prescribing practices tended to change their course (data not shown).

The solo practices had larger prescribing variations than group practices (0.30 SD units, 95% CI 0.24–0.35, versus 0.19, 95% CI 0.15–0.23; *P* < 0.005). Among the solo practices there was no difference in prescribing variation between male and female GPs, but the variation decreased with 0.02 SD units by year of GP age. There were no significant relationships between age, sex, and number of years as GP on the one hand and prescribing volume over time on the other (data not shown).

## Discussion

There was a considerable variation in prescribing levels between practices but a considerable stability in the variation of prescribing behaviour over the study period for individual practices, irrespective of analysis method. The mailed feedback had no detectable effect on prescribing behaviour. As expected, there was slightly more variation within solo practices than within group practices because of the counterbalancing effect of accumulated prescribing by of two or more GPs in the group practices. The variation decreased somewhat with GP age, and there were no statistically significant gender effects.

The analyses were performed on official data, based on filled prescriptions, with little or no data loss. The same authority registered all filled prescriptions, minimizing handling variation. Potential disturbing factors, such as auto-correlation, were eliminated during data processing. It was not possible to establish a control group within the county, as the quality improvement initiative (‘GP Quality Unit’) aimed at covering all practices. Comparison with other counties was not possible, since data were not available for areas other than Storstrøm County. Therefore, the practices served as their own controls over time. Data refer to prescribing habits during the 1990s. However, the problem of rational prescribing habits is still prevalent (11).

Few authors have addressed the issue of stability of prescribing habits in general practice. One of the main reasons may be the lack of comprehensive long-term prescription data registers at prescribing physician level. In a New Zealand study from 1992–94 (5), based on reimbursement data, a 9% median intra-GP variability was found in both volume and total costs from year to year in a regional GP sample (305 GPs), and a 16% variation in total costs and 17% in total volume in a national GP sample (74 GPs).

Our finding of no effects of mailed feedback on GPs' prescribing behaviour conforms to what has been shown in a comprehensive 2000 Cochrane review (6). Similar results have also been shown in the few studies where large data registers were used to collect outcome measures. In a randomized controlled trial in Australia (7), no effects were found of unsolicited, posted government-sponsored feedback based on centralized aggregated data on prescribing levels of general practitioners. In a Danish randomized, controlled trial it was concluded that postal prescribing feedback in addition to clinical guidelines on the diagnosis and treatment of respiratory tract infections did not influence GPs' prescribing patterns (8,9).

However, there are studies that demonstrate some effects. In a Canadian trial 54 GPs whose prescribing of analgesics was more than two standard deviations above average were randomly allocated to receiving a note on their prescribing volume and a 1-day group education activity, or to receiving a written notification only, or to no intervention (14). Those in the first group decreased their prescribing volume by 33%, and those in the second group by 25%, while there was no change in the third group. Similar but smaller effects were found in a Norwegian study (10) and in a Canadian study (11) when written feedback of prescribing profiles was combined with treatment recommendations. In a 2006 Cochrane review it vwas found that the combination of audit and feedback had a small to moderate effect on professional practice (15).

Although mailed feedback only has shown a modest or no effect on doctors' drug prescribing, it is still widely used in continuing medical education (CME) and in quality assurance and improvement. The method is easy to apply on a large scale and relatively cheap. However, the approach appears to be more effective if combined with other strategies (16), such as audit feedback with peer discussions (17,18).

Some possible explanations for the lack of success with mailed feedback only in this study might be that the GPs may not have paid attention to the diagrams, or they may not have understood the diagrams, or they may have taken the diagrams into consideration but found no reason to chance their prescribing habits. Moreover, too much information with poor explanation may have been provided.

It is important to note that the establishing of the ‘GP Quality Unit’, the development of the feedback charts, and the semi-annual mailed prescribing feedback were some of the first, but important, steps in the process of establishing a formal local quality improvement culture within the general practice community of Storstrøm County in the early 1990s. The main purpose of the initiative was to initiate reflections on variations in prescribing behaviour and raise awareness about prescribing patterns. The feedback diagrams were not accompanied by any clinically relevant information, and they were based on aggregated prescribing data at the second ATC level with no chance of identifying specific substances at the fifth ATC level. On the other hand, too detailed prescribing information at this early stage in the quality assurance process might possibly impede the overall ambition of starting a debate about rational drug prescribing in a broader sense.

## Conclusions

No apparent effect of mailed feedback on prescribing habits in general practice was found. Other, more activating approaches than postal feedback may be necessary to affect GPs' prescribing behaviour.

## References

[1] Swedish Pharmacy Authority http://www.apoteket.se/rd/d/2835.

[2] (1979). Nordic Statistics on Medicines 1975–1977. Statistical reports of the Nordic Countries no 35, 36.

[3] Maronde RF, Lee PV, McCarron MM, Seibert S (1971). A study of prescribing patterns. Med Care.

[4] Oxman AD, Thomson MA, Davis DA, Haynes RB (1995). No magic bullets: a systematic review of 102 trials of interventions to improve professional practice. CMAJ.

[5] Bishop N, Maling T (2000). Variability within general practitioner prescribing over time. N Z Med J.

[6] Freemantle N, Harvey EL, Wolf F, Grimshaw JM, Grilli R, Bero LA (2000). Printed educational materials: effects on professional practice and health care outcomes. Cochrane Database Syst Rev.

[7] O'Connell DL, Henry D, Tomlins R (1999). Randomised controlled trial of effect of feedback on general practitioners' prescribing in Australia. BMJ.

[8] Sondergaard J, Andersen M, Stovring H, Kragstrup J (2003). Mailed prescriber feedback in addition to a clinical guideline has no impact: a randomised, controlled trial. Scand J Prim Health Care.

[9] Sondergaard J, Andersen M, Vach K, Kragstrup J, Maclure M, Gram LF (2002). Detailed postal feedback about prescribing to asthma patients combined with a guideline statement showed no impact: a randomised controlled trial. Eur J Clin Pharmacol.

[10] Rokstad K, Straand J, Fugelli P (1995). Can drug treatment be improved by feedback on prescribing profiles combined with therapeutic recommendations? A prospective controlled trial in general practice. J Clin Epidemiol.

[11] Herbert CP, Wrigtht JM, Maclure M, Wakefield J, Dormuth C, Brett-MacLean P (2004). Better Prescribing Project: a randomized controlled trial of the impact of case-based educational modules and personal prescribing feedback on prescribing for hypertension in primary care. Fam Pract.

[12] WHO Collaborating Centre for Drug Statistics Methodology (1976). The Anatomical Therapeutic Chemical Classification System.

[13] Statistical Analysis System http://www.sas.com/technologies/analytics/statistics/ /factsheet.pdf.

[14] Anderson JF, McEwan KL, Hrudey WP (1996). Effectiveness of notification and group education in modifying prescribing of regulated analgesics. CMAJ.

[15] Jamtvedt G, Young JM, Kristoffersen DT, O'Brien MA, Oxman AD (2006). Audit and feedback: effects on professional practice and health care outcomes. Cochrane Database Syst Rev.

[16] Wensing M, Grol R (1994). Single and combined strategies for implementing changes in primary care: a literature review. Int J Qual Health Care.

[17] Wahlstrom R, Kounnavong S, Sisounthone B, Phanyanouvong A, Southammavong T, Eriksson B (2003). Effectiveness of feedback for improving case management of malaria, diarrhoea and pneumonia—a randomized controlled trial at provincial hospitals in Lao PDR. Trop Med Int Health.

[18] Nilsson G, Hjemdahl P, Hassler A, Vitols S, Wallen NH, Krakau I (2001). Feedback on prescribing rate combined with problem-oriented pharmacotherapy education as a model to improve prescribing behaviour among general practitioners. Eur J Clin Pharmacol.

